# Phylogeography of *Bacillus anthracis* in the Country of Georgia Shows Evidence of Population Structuring and Is Dissimilar to Other Regional Genotypes

**DOI:** 10.1371/journal.pone.0102651

**Published:** 2014-07-21

**Authors:** Ekaterine Khmaladze, Dawn N. Birdsell, Amber A. Naumann, Christian B. Hochhalter, Meagan L. Seymour, Roxanne Nottingham, Stephen M. Beckstrom-Sternberg, James Beckstrom-Sternberg, Mikeljon P. Nikolich, Gvantsa Chanturia, Ekaterine Zhgenti, Mariam Zakalashvili, Lile Malania, Giorgi Babuadze, Nikoloz Tsertsvadze, Natalia Abazashvili, Merab Kekelidze, Shota Tsanava, Paata Imnadze, Holly H. Ganz, Wayne M. Getz, Ofori Pearson, Pawel Gajer, Mark Eppinger, Jacques Ravel, David M. Wagner, Richard T. Okinaka, James M. Schupp, Paul Keim, Talima Pearson

**Affiliations:** 1 National Center for Disease Control and Public Health, Tbilisi, Georgia; 2 Center for Microbial Genetics and Genomics, Northern Arizona University, Flagstaff, Arizona, United States of America; 3 Walter Reed Army Institute of Research, Silver Spring, Maryland, United States of America; 4 Department Environmental Science Policy and Management, University of California, Berkeley, California, United States of America; 5 US Geological Survey, Denver Federal Center, Denver, Colorado, United States of America; 6 Institute for Genome Sciences, Baltimore, Maryland, United States of America; 7 University of Texas at San Antonio, Texas, United States of America; 8 Ivane Javakhishvili Tbilisi State University, Tbilisi, Georgia; Loyola University Medical Center, United States of America

## Abstract

Sequence analyses and subtyping of *Bacillus anthracis* strains from Georgia reveal a single distinct lineage (Aust94) that is ecologically established. Phylogeographic analysis and comparisons to a global collection reveals a clade that is mostly restricted to Georgia. Within this clade, many groups are found around the country, however at least one subclade is only found in the eastern part. This pattern suggests that dispersal into and out of Georgia has been rare and despite historical dispersion within the country, for at least for one lineage, current spread is limited.

## Introduction

*Bacillus anthracis*, the causative agent of anthrax, continues to decimate livestock herds and cause concern over possible nefarious use. Source attribution of outbreaks is therefore essential for epidemiological and forensic investigations as well as control efforts. Accurate attribution requires determining evolutionary relationships among isolates and understanding how they fit into regional and global phylogeographic patterns. Genetic characterization of *B. anthracis* first gained considerable traction through multiple locus VNTR analysis (MLVA) [1] which identified major genetic groups. MLVA typing schemes have been expanded to include more loci; however, although these methods provide additional resolution, inferences on relationships among isolates are unreliable due to the prevalence of convergent alleles, negatively affecting the accuracy of downstream attribution conclusions. With the increased accessibility of whole genome sequencing, typing schemes based on single nucleotide polymorphisms (SNPs) provide both high resolution and highly accurate phylogenetic information [2]–[4] although phylogenetic discovery bias [2] must be taken into account.

We and others have previously used MLVA and SNP typing to characterize regional and global phylogeographic patterns of *B. anthracis*. Although ungulate grazers certainly play a role in the dissemination of this species, human-mediated dispersal has contributed to both ancient [5] and recent [6] spread. Contaminated animal products are frequently identified during outbreaks of disease in humans [6], [7]; however, ecological establishment of exotic strains is rare [4], [8]. None the less, distinguishing between endemic and non-indigenous strains is difficult and can obscure phylogeographic patterns and confuse attribution efforts. Intensive regional sampling studies are not only invaluable for identifying endemic strains and for defining regional patterns of dissemination and cycling, but also build the foundation for understanding global patterns of spread.

We report here the phylogeographic patterns of *B. anthracis* samples collected within the country of Georgia. Located in a geographic bottleneck between the Black and Caspian Seas, and between Europe and Asia, this region has been impacted by ancient and modern human movement and trade. Such anthropogenic influences may have shaped the distribution of *B. anthracis* in this region and may prove to be important in understanding global phylogeographic patterns.

## Materials and Methods

### Phylogenetic placement

To place the 272 Georgian isolates into the established global phylogeny [2], [6]–[8], we screened all isolates with previously described canSNP assays [8]. All isolates were assigned to the Aust94 genetic group. To obtain more detailed resolution within this genetic group, we genotyped the Georgian isolates and 225 additional Aust94 group isolates from our global collection with previously published Aust94 assays [9](Table S1) as well as with novel assays (see CanSNP Selection and Analysis below). DNA templates were extracted using either chloroform [10], DNeasy blood and tissue kits (Qiagen, Valencia, CA) or heat soak.

### Whole Genome Sequencing

To further resolve the genetic structure within Georgian Aust94 group, we sequenced the genomes of three Georgian isolates belonging to the Aust94 genetic group, using Illumina's Genome Analyzer II (San Diego, CA). Library preparation for this isolate involved sonication of 5 µg genomic DNA, obtained through a standard chloroform extraction protocol [10] and shearing the DNA to an average fragment size of 350 bp. The library was quantified using SYBR-based qPCR and primers modified from the adaptor sequence. Paired-end read lengths were ∼100 bp. The sequences of 52-G, 9080-G, and 8903-G were deposited into GenBank (PRJNA224563, PRJNA224558, and PRJNA224562, respectively) (Table S2).

### SNP Discovery and Analysis

**Figure 1:**
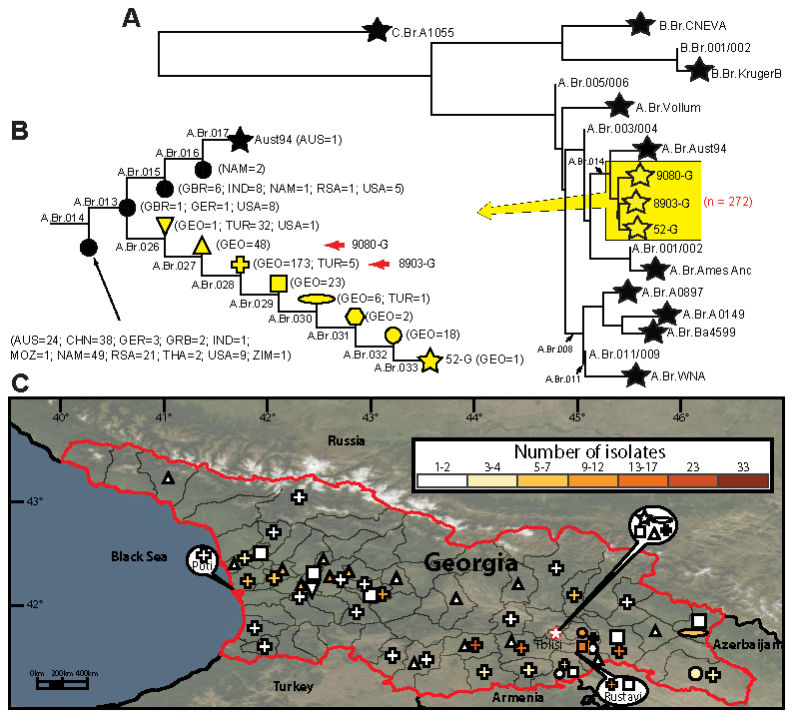
*Bacillus anthracis* phylogenetics in Georgia. A) Established phylogeny of *B. anthracis* [2], [6]–[8]. Terminal subgroups representing sequenced strains are shown as stars, and intervening nodes representing collapsed branches appear as circles. The highlighted yellow box (part of the Aust94 lineage) indicate the phylogenetic location of Georgian strains. Stars within the highlighted yellow area represent the three Georgian strains sequenced for this study. The number of Georgian strains is indicated in red. B) Expansion of the Aust94 group and canSNP subgroups within the Georgian lineage. The number and origin of isolates are shown for each node and stars or red arrows indicate the locations of the sequenced strains. Shapes of nodes correspond to geographic location. (C) Phylogeography of 272 *B. anthracis* isolates falling in the Aust94 group are mapped across the country of Georgia at a district level. The heat map legend indicates the number of isolates per subgroup found in a given district.

To identify putative SNPs, homologous genomic regions were identified using MUMmer [11] and aligned to search for SNPs using SolSNP (http://sourceforge.net/projects/solsnp/). We ensured site orthology by eliminating potential paralogs and requiring all genome alignments to include 100 bp flanking each side of the SNP. Furthermore, for analysis inclusion, all SNP loci were required to be present in all of the genomes analyzed. A maximum-parsimony tree was constructed by PAUP 4.0b10 software (Sinauer Associates, Inc., Sunderland, MA, USA) using all putative SNPs from this study and ten published genomes (Figure 1, panel A; Table S2).

### CanSNP Selection and Analysis

WGS comparisons between Georgian (52-G) and Aust94 (Aust94, AAES00000000) strains revealed 50 putative SNPs specific to 52-G. Of these, twenty-six were incorporated into melt-MAMA genotyping assays, as previously described [9] and eight were selected as canonical SNP assays (Table 1). Allele-specific melt-MAMA primers were designed using Primer Express 3.0 software (Applied Biosystems, Foster City, CA). All other assay reagents and instrumentation were as previously described [9]. PCR reactions were first raised to 50°C for 2 min to activate the uracil glycolase, then raised to 95°C for 10 min to denature the DNA and then cycled at 95°C for 15 s and 55°C–60°C for 1 min for 33 cycles (Table 1). Immediately after the completion of the PCR cycle, amplicon melt dissociation was measured by ramping from 60°C to 95°C in 0.2°C/min increments and recording the fluorescent intensity.

**Table 1:** Melt-MAMA primers targeting canonical SNPs for 8 new phylogenetic branches discovered in this study^a^.

Branch	AmesAnc position^b^	Genome SNP state (D/A)^c^	Melt-MAMA primer sequence^d^	Conc, µM^e^	Annealing temp °C
A.Br.026	3,640,599	T/C	A:CTTCTTTTAATACATCTAAGTAAGTAAGCGTTgC D:cggggcggggcggggcggggCTTCTTTTAATACATCTAAGTAAGTAAGCGTTcTC:ATTGACCCAACAGCTACGAAATAC	0.45 0.15 0.15	60
A.Br.027	4,355,524	A/G	A:CCCATTCCAAGTGACACACTcG D:cggggcggggcggggcggggCCCATTCCAAGTGACACACTgA C:AGCACTTGCTTATCTTGGAGCTT	0.60 0.15 0.15	60
A.Br.028	791,256	A/G	A:ACAGAGAAGGTTATAAGTCCAGAcGG D:cggggcggggcggggcggggACAGAGAAGGTTATAAGTCCAGAaGA C:CTCGCTTTTCCTGTTCTTTTATTCAC	0.15 0.15 0.15	60
A.Br.029	3,960,657	A/G	A:AGTATTCCAACCATTACTATAGTCACTaG D:ggggcggggcggggcggggcggggcAGTATTCCAACCATTACTATAGTCACTcAC:GTACTTATTGGTGGTACTGCCAAATT	0.15 0.15 0.15	60
A.Br.030	3,528,668	A/G	A:CAATCCCTCGATTTACATATAAATATAAaG D:ggggcggggcggggcggggcggggcCAATCCCTCGATTTACATATAAATATAAcAC:AGGTATGTATGAATTAGAAGGGAAGAA	0.15 0.15 0.15	60
A.Br.031	3,018,054	C/T	A:ACTATCGCCAAAAGCAATTGaAT D:cggggcggggcggggcggggACTATCGCCAAAAGCAATTGtAC C:TATTTTAGACAAGTACGAACTAGATAAATCAA	0.15 0.15 0.15	55
A.Br.032	3,520,170	G/A	A:CCACCAACAACGAATGGAAGtA D:cggggcggggcggggcggggCCACCAACAACGAATGGAAGaG C:AGCATTTAATGAACGGCGTAAGTAATA	0.45 0.15 0.15	60
A.Br.033	3,610,151	C/T	A:TAAATAACCAAGGCGTCTTGCCAT D:ggggcggggcggggcggggcggggcCTAAATAACCAAGGCGTCTTGCtAC C:TGTAGGACGTAGTATGGTGAAAGTAGTAGAT	0.60 0.15 0.15	60

a Melt-MAMA, melt-mismatch amplification mutation assay; SNP, single nucleotide polymorphism; con, concentration.

b Ames Ancestor reference genome (NC_006570).

c SNP states are presented according to the top strand in the Ames ancestor AE017334 D: Derived SNP state; A: Ancestral SNP state.

d Melt-mismatch amplification mutation assay (MAMA), A: Ancestral; D: Derived; C: Common. Primer tails and antepenultimate or penultimate mismatch bases are in lower case.

e Final concentratinon of each primer in Melt-MAMA genotyping assays.

## Results and Discussion

Screening of 272 Georgian isolates with previously described canSNP assays [9] resulted in the assignment of all isolates to the group defined by the Australia94 (Aust94) genome [8] (Figure 1, panel A). The temporal, geographic and phylogenetic diversity of the Georgian Aust94 isolates, coupled with the detection of MLVA A3a [1] (roughly equivalent to the Aust94 lineage) isolates in the region by other researchers [12]–[15] is strong evidence for the ecological establishment of this clade (Table S3). Reference isolates from Turkey in the MLVA A3a group reported in Keim et al. [1] include six genotypes (genotypes 33, 36, 37, 41–43) and thus show regional diversity that is also reflected by the SNP genotyping here.

Members of the Aust94 group have been identified on five continents (Figure 1, panel B), suggesting extensive dispersal [8], however detailed phylogeography of any part of this lineage has not been previously described. The resulting whole genome SNP phylogenetic tree (Figure 1, panel A) (Table S2) drawn from 13 strains placed the three sequenced Georgian genomes within the Aust94 genetic group. Screening our global collection across previously published SNP assays (11) revealed five genetic groups along the Aust94 lineage (Figure 1, panel B) (Table S1 and S3) and eight novel groups along the lineage terminating in the 52-G genome.

The branches and topology leading to isolates that were genotyped, but not sequenced, remain unknown due to phylogenetic discovery bias and branch collapse, however clade membership is accurately estimated [2], [4]. The node between branches A.Br.014 and A.Br.013 (named A.Br.014/013) forms the most basal subgroup and contains isolates collected from countries in five continents (Figure 1, panel B). The A.Br.013/015 subgroup contains isolates from Europe (n = 2) and USA (n = 8). It also gives rise to the lineages that contain all 272 Georgian isolates (Figure 1, panel B) as well as the lineage leading to the Aust94 genome. Despite the identification of isolates within this A.Br.013/015 node from Europe and the USA, this genetic group is not ecologically established in these regions and is thus not likely to be the source of the introduction into the Georgia/Turkey region. Rather, the presence of these isolates in Europe and the USA is likely to be due to the importation of contaminated animal products, possibly from the same geographic region responsible for the introduction into the Georgia/Turkey region. Additional phylogeography studies of these basal lineages are needed identify this source.

To further resolve the A.Br.013/015 group to understand the Georgian population structure, we screened all members of this group (including the 272 Georgian isolates) across the twenty-six SNP assays leading out to the 52-G strain, resulting in the identification of eight new groups within this lineage. All Georgian isolates fell within one of the eight new groups (Figure 1, panel B)(Table S3); however, some isolates from Turkey and one from the USA were also placed within these groups. The single USA isolate is probably from a contaminated animal product imported from the Turkey/Georgia region. Most Turkish isolates included in this study are assigned to the basal node along the 52-G lineage. However, as we have no knowledge of the phylogenetic topology within this basal node, it is impossible to determine if this group was first introduced into Turkey and subsequently dispersed into Georgia or *vice versa*; both scenarios are equally parsimonious. The presence of Turkish isolates in two more recent nodes preceded by exclusively Georgian nodes suggests at least two dispersal events from Georgia to Turkey (Figure 1, panel B). Further sampling from neighboring countries will provide more details on the geographic limits of this 52-G clade and the impact of national boundaries on limiting the dispersal of *B. anthracis* in the area.

In any clade, members of the more ancient nodes have the greatest potential for widespread geographic dispersion. Indeed, along the lineage to the 52-G genome, members of the more basal nodes with multiple isolates from Georgia have been isolated across the country (Figure 1, panels B and C). However, without further sequencing of representative strains within each node, the phylogenetic topology cannot be known due to branch collapse [2]. Without phylogenetic knowledge, it is not possible to determine if phylogeographic clustering occurs at more recent evolutionary levels within these nodes. Conversely, isolates belonging to the clade after A.Br.030 which includes three nodes and the 52-G genome are only found in the southeast of Georgia (Figure 1, panel C), indicating more modern restrictions to dispersal. It is therefore likely that similar geographic structuring exists within the more basal nodes and would indicate that current dispersal of *B. anthracis* around Georgia is rare.

## Conclusions

Our results are consistent with complex global dispersal patterns that have resulted in worldwide dispersal of the Aust94 group [6]–[8]. This work now provides additional resolution and detail within the Aust94 group and shows it to be highly geographically structured with a group of closely related isolates being largely restricted to the region in and around Georgia and Turkey. Even in Georgia there is evidence of geographic structuring within the one lineage we characterized in detail, suggesting that that although anthrax has been dispersed throughout the country, current dispersal may be limited.

## Supporting Information

Table S1 **Melt-MAMAs published in Birdsell et al 2012 and used in this study.**
(XLSX)

Table S2 **Reference strains plus three new genomes used in this study and their NCBI accession numbers.**
(XLSX)

Table S3 **Epidemiological and genotyping data for all** ***B. anthracis*** **strains included in this study.**
(XLSX)
